# Geometry of color perception. Part 2: perceived colors from real quantum states and Hering’s rebit

**DOI:** 10.1186/s13408-020-00092-x

**Published:** 2020-09-09

**Authors:** M. Berthier

**Affiliations:** Laboratoire MIA, La Rochelle Université, Avenue Albert Einstein, BP 33060, 17031 La Rochelle, France

**Keywords:** Color perception, Quantum states, Jordan algebras, Quantum rebit

## Abstract

Inspired by the pioneer work of H.L. Resnikoff, which is described in full detail in the first part of this two-part paper, we give a quantum description of the space $\mathcal{P}$ of perceived colors. We show that $\mathcal{P}$ is the effect space of a rebit, a real quantum qubit, whose state space is isometric to Klein’s hyperbolic disk. This chromatic state space of perceived colors can be represented as a Bloch disk of real dimension 2 that coincides with Hering’s disk given by the color opponency mechanism. Attributes of perceived colors, hue and saturation, are defined in terms of Von Neumann entropy.

## Introduction

“The structure of our scientific cognition of the world is decisively determined by the fact that this world does not exist in itself, but is merely encountered by us as an object in *the correlative variance of subject and object*” [1].

### On the mathematics of color perception

The mathematical description of human color perception mechanisms is a longstanding problem addressed by many of the most influential figures of the mathematical physics [1–4]. The reader will find an overview of the main historical contributions at the beginning of [5] where H.L. Resnikoff points out that the space, which we denote by ${\mathcal{P}}$, of perceived colors is one of the very first examples of abstract manifold mentioned by B. Riemann in his habilitation [6], “a pregnant remark”. As suggested by H. Weyl [1], it is actually very tempting to characterize the individual color perception as a specific correlative interaction between an abstract space of perceived colors and an embedding space of physical colors. This raises the question of defining intrinsically, in the sense of Riemannian geometry, the space of perceived colors from basic largely accepted axioms. These axioms, which date back to the works of H.G. Grassmann and H. Von Helmholtz [2, 7], state that ${\mathcal{P}}$ is a regular convex cone of dimension 3. It is worth noting that convexity reflects the property that one must be able to perform mixtures of perceived colors or, in other words, of color states [8]. What makes the work of H.L. Resnikoff [5] particularly enticing is the remarkable conclusions that he derives by adding the sole axiom that ${\mathcal{P}}$ is homogeneous under the action of the linear group of background illumination changes [9]. We will discuss in Sect. 6 the relevance of this statement. To the best of our knowledge, this axiom, which involves an external context, has never been verified by psychophysical experiments. It endows ${\mathcal{P}}$ with the rich structure of a symmetric cone [10]. With this additional axiom, and the hypothesis that the distance on ${\mathcal{P}}$ is given by a Riemannian metric invariant under background illumination changes, H.L. Resnikoff shows that ${\mathcal{P}}$ can only be isomorphic to one of the two following Riemannian spaces: the product $\mathcal{P}_{1}=\mathbb{R}^{+}\times \mathbb{R}^{+}\times \mathbb{R}^{+}$ equipped with a flat metric, namely the Helmholtz–Stiles metric [11], and $\mathcal{P}_{2}=\mathbb{R}^{+}\times \operatorname{SL}(2,\mathbb{R})/ \operatorname{SO}(2,\mathbb{R})$ equipped with the Rao–Siegel metric of constant negative curvature [12, 13]. Let us recall that the quotient $\operatorname{SL}(2,\mathbb{R})/\operatorname{SO}(2,\mathbb{R})$ is isomorphic to the Poincaré hyperbolic disk $\mathcal{D}$. The first space is the usual metric space of the colorimetry, while the second one seems to be relevant to explain psychophysical phenomena such as the ones described by H. Yilmaz in [14] and [15] or physiological mechanisms such as the neural coding of colors of R. and K. de Valois [16, 17]. In the sequel, we focus on the latter.

### A quantum glance at color perception

The starting point of this work originates from the second part of [5] dedicated to Jordan algebras. Contrary to H.L. Resnikoff we suppose at first that the perceived color space ${\mathcal{P}}$ can be described from the state space of a quantum system characterized by a Jordan algebra ${\mathcal{A}}$ of real dimension 3 [18–21]. This is our only axiom, see Sect. 2.1 for motivations. Jordan algebras are non-associative commutative algebras that have been classified by P. Jordan, J. Von Neumann, and E. Wigner [22] under the assumptions that they are of finite dimension and formally real. They are considered as a fitting alternative to the usual associative noncommutative algebraic framework for the geometrization of quantum mechanics [23–25]. Not so surprisingly in view of what precedes, $\mathcal{A}$ is necessarily isomorphic to one of the two following Jordan algebras: the algebra $\mathbb{R}\oplus \mathbb{R}\oplus \mathbb{R}$ or the algebra $\mathcal{H}(2,\mathbb{R})$ of symmetric real 2 by 2 matrices. It appears that the two geometric models of H.L. Resnikoff can be recovered from this fact by simply taking the positive cone of $\mathcal{A}$. The Jordan algebra $\mathcal{H}(2,\mathbb{R})$ carries a very special structure being isomorphic to the spin factor $\mathbb{R}\oplus \mathbb{R}^{2}$. It can be seen as the non-associative algebra linearly spanned by the unit 1 and a spin system of the Clifford algebra of $\mathbb{R}^{2}$ [21, 26]. The main topic of this work is to exploit these structures to highlight the quantum nature of the space $\mathcal{P}$ of perceived colors. Actually, the quantum description that we propose gives a precise meaning to the relevant remark of [27], p. 539: “This underlying mathematical structure^1^ is reminiscent of the structure of states (i.e., density matrices) in quantum mechanics. The space of all states is also convex-linear, the boundary consists of pure states, and any mixed state can be obtained by a statistical ensemble of pure states. In the present case, the spectral colors are the analogs of pure states”.

Although the geometry of the second model $\mathcal{P}_{2}$ of H.L. Resnikoff is much richer than the geometry of the first model $\mathcal{P}_{1}$, very few works are devoted to the possible implications of hyperbolicity in color perception. One of the main objectives of this contribution is to show that the model $\mathcal{P}_{2}$ is perfectly adapted to explain the coherence between the trichromatic and color opponency theories. We show that the space $\mathcal{P}$ is the effect space of a so-called rebit, a real quantum qubit, whose state space $\mathcal{S}$ is isometric to the hyperbolic Klein disk $\mathcal{K}$. Actually, $\mathcal{K}$ is isometric to the Poincaré disk $\mathcal{D}$, but its geodesics are visually very different, being the straight chords of the unit disk. Klein geometry appears naturally when considering the spin factor $\mathbb{R}\oplus \mathbb{R}^{2}$ and the 3-dimensional Minkowski future lightcone $\mathcal{L}^{+}$ whose closure is the state cone of the rebit. We show that the chromatic state space $\mathcal{S}$ can be represented as a Bloch disk of real dimension 2 that coincides with the Hering disk given by the color opponency mechanism. This Bloch disk is an analog, in our real context, of the Bloch ball that describes the state space of a two-level quantum system of a spin-$\frac{1}{2}$ particle. The dynamics of this quantum system can be related to the color information processing that results from the activity rates of the four types of spectrally opponent cells [17], see Sect. 7.1. The spectrally opponent interactions in primates are usually considered to be performed by ganglion and lateral geniculate nucleus cells which are very similar with regards to color processing [17, 28].

Following this quantum interpretation, we give precise definitions of the two chromatic attributes of a perceived color, hue and saturation, in terms of Von Neumann entropy.

As explained by P.A.M. Dirac in [29], p. 18, physical phenomena justify the need for considering complex Hilbert spaces in quantum mechanics. Alternatively, the structures we deal with in the sequel are real, and we may consider the space $\mathcal{P}$ as a nontrivial concrete example of an effect space of a real quantum system. The reader will find more information on real-vector-space quantum theory and its consistency regarding optimal information transfer in [30].

Finally, since the spin factors and the corresponding Clifford algebras share the same representations (and the same squares), one may envisage to adapt to the present context the tools developed in [31] for the harmonic analysis of color images.

### Outline of the paper

We introduce in Sect. 2 the mathematical notions that are used to recast the description of the perceived color space geometry into the quantum framework. We begin by explaining the motivations and meaning of the trichromacy axiom which is the cornerstone of our approach. Section 3 is devoted to quantum recalls. It mainly contains the material needed to describe the state space of the so-called rebit, i.e., the two-level real quantum system. Section 4 contains results on Riemannian geometry. The objective of this section is to show that Klein’s geometry, or equivalently Hilbert’s geometry, is well adapted to quantum states, contrary to the Poincaré geometry used by H.L. Resnikoff. We propose in Sect. 5 to interpret perceived colors as quantum measurement operators. This allows us in particular to give mathematically sound colorimetric definitions. Section 6 is devoted to some results on group actions and homogeneity in relation with the supplementary axiom of H.L. Resnikoff. Finally, in Sect. 7, we discuss some consequences of our work in relation with the neural color coding and relativistic models of respectively R. and K. de Valois, and H. Yilmaz. We also point out some perspectives regarding the links between MacAdam ellipses and Hilbert’s metric.

## Mathematical preliminaries

We introduce in this section the mathematical notions needed in the sequel. They mainly concern the properties of Jordan algebras. The reader will find more detailed information on this subject in [18, 20, 21, 32] or in the seminal work of P. Jordan [33].

### The trichromacy axiom

Before going into detail, we give some explanations in order to justify the mathematical approach adopted. Following the axioms of H.G. Grassmann and H. Von Helmholtz [2, 7], the space of perceived colors is a regular convex cone of real dimension 3. Such a geometrical structure does not carry enough information to allow relevant developments. The main idea of Resnikoff’s work is to enrich this structure by requiring that the cone of perceived colors be homogeneous [9]. It appears that if we add one more property, namely self-duality, this cone becomes a symmetric cone [10]. The fundamental remark is that being symmetric the cone of perceived colors can be considered as the set of positive observables of a formally real Jordan algebra $\mathcal{A}$. This is precisely the statement of the famous Koecher–Vinberg theorem [32]. Since the cone is of real dimension 3, the algebra is also of real dimension 3. Using the classification theorem of P. Jordan, J. Von Neumann, and E. Wigner [22], one can check that the algebra $\mathcal{A}$ is necessarily isomorphic either to the Jordan algebra $\mathbb{R}\oplus \mathbb{R}\oplus \mathbb{R}$ or to the Jordan algebra $\mathcal{H}(2,\mathbb{R})$ of symmetric real 2 by 2 matrices. In consequence, adding the self-duality property, Resnikoff’s classification stems from the classification theorem of P. Jordan, J. Von Neumann, and E. Wigner.

The point of view that we wish to put forward is that perceived colors must be described by measurements through some state-observable correspondence. This is formalized in Sect. 5.1 with the notion of quantum effects. In order to emphasize this point of view, we formulate our starting trichromacy axiom as follows: *the Grassmann–Von Helmholtz cone of perceived colors is the positive cone of a formally real Jordan algebra of real dimension* 3*.*

### Jordan algebras and symmetric cones

A Jordan algebra $\mathcal{A}$ is a real vector space equipped with a commutative bilinear product $\mathcal{A}\times \mathcal{A}\longrightarrow \mathcal{A}$, $(a,b)\longmapsto a\circ b$, satisfying the following Jordan identity:

1 $$ \bigl(a^{2}\circ b\bigr)\circ a=a^{2}\circ (b\circ a) . $$

This Jordan identity ensures that the power of any element *a* of $\mathcal{A}$ is well defined ($\mathcal{A}$ is power associative in the sense that the subalgebra generated by any of its elements is associative). Since a sum of squared observables never vanishes, one logically requires that if $a_{1}, a_{2},\ldots, a_{n}$ are elements of $\mathcal{A}$ such that

2 $$ a_{1}^{2}+a_{2}^{2}+\cdots +a_{n}^{2}=0 , $$

then $a_{1}=a_{2}=\cdots =a_{n}=0$. The algebra $\mathcal{A}$ is then said to be formally real. This property endows $\mathcal{A}$ with a partial ordering: $a\leq b$ if and only if $b-a$ is a sum of squares, and therefore the squares of $\mathcal{A}$ are positive. Formally real Jordan algebras of finite dimension are classified [22]: every such algebra is the direct sum of so-called simple Jordan algebras. Simple Jordan algebras are of the following types: the algebras $\mathcal{H}(n,\mathbb{K})$ of hermitian matrices with entries in the division algebra $\mathbb{K}$ with $\mathbb{K}=\mathbb{R}$, $\mathbb{C}$, $\mathbb{H}$ (the algebra of quaternions), the algebra $\mathcal{H}(3,\mathbb{O})$ of hermitian matrices with entries in the division algebra $\mathbb{O}$ (the algebra of octonions), and the spin factors $\mathbb{R}\oplus \mathbb{R}^{n}$, with $n\geq 0$. The Jordan product on $\mathcal{H}(n,\mathbb{K})$ and $\mathcal{H}(3,\mathbb{O})$ is defined by

3 $$ a\circ b=\frac{1}{2}(ab+ba) . $$

Note that contrary to the usual matrix product, this product is effectively commutative. The spin factors form “the most mysterious of the four infinite series of [simple] Jordan algebras” [34]. They were introduced for the first time under this name by D.M. Topping [35] and are defined as follows. The spin factor $J(V)$ of a given *n*-dimensional real inner product space *V* is the direct sum $\mathbb{R}\oplus V$ endowed with the Jordan product

4 $$ (\alpha +\mathbf{v})\circ (\beta +\mathbf{w})=\bigl(\alpha \beta +\langle { \mathbf{v}},{ \mathbf{w}}\rangle +\alpha {\mathbf{w}}+\beta {\mathbf{v}}\bigr) , $$

where *α* and *β* are reals, and **v** and **w** are vectors of *V*. The following result is well known [34].

#### Proposition 1

*Let* $\mathbb{K}$ *be the division algebra* $\mathbb{R}$, $\mathbb{C}$, $\mathbb{H}$, *or* $\mathbb{O}$. *The spin factor* $J(\mathbb{K}\oplus \mathbb{R})$ *is isomorphic to the Jordan algebra* $\mathcal{H}(2,\mathbb{K})$.

#### Proof

An explicit isomorphism is given by the map

5 $$\begin{aligned}& \phi : \mathcal{H}(2,\mathbb{K})\longrightarrow J(\mathbb{K}\oplus \mathbb{R}), \end{aligned}$$

6 $$\begin{aligned}& \begin{pmatrix} \alpha +\beta & x \\ x^{*} & \alpha -\beta \end{pmatrix}\longmapsto (\alpha +x+\beta ) , \end{aligned}$$

with *x* in $\mathbb{K}$ and $x^{*}$ the conjugate of *x*. □

Now, we focus on the algebra $\mathcal{H}(2,\mathbb{R})$ and the spin factor $J(\mathbb{R}\oplus \mathbb{R})$, both being isomorphic to $\mathbb{R}\oplus \mathbb{R}^{2}$. The latter is equipped with the Minkowski metric [36]

7 $$ (\alpha +\mathbf{v})\cdot (\beta +\mathbf{w})=\alpha \beta -\langle { \mathbf{v}},{ \mathbf{w}}\rangle , $$

where *α* and *β* are reals, and **v** and **w** are vectors of $\mathbb{R}^{2}$. It turns out that Proposition 1 has a fascinating reformulation: the algebra of observables of a 2-dimensional real quantum system is isomorphic to the 3-dimensional Minkowski spacetime. Let us recall that the lightcone ${\mathcal{L}}$ of $\mathbb{R}\oplus \mathbb{R}^{2}$ is the set of elements $a=(\alpha +\mathbf{v})$ that satisfy

8 $$ a\cdot a=0 , $$

and that a light ray is a 1-dimensional subspace of $\mathbb{R}\oplus \mathbb{R}^{2}$ spanned by an element of ${\mathcal{L}}$. Every such light ray is spanned by a unique element of the form $(1+\mathbf{v})/2$ with **v** a unit vector of $\mathbb{R}^{2}$. Actually, the space of light rays coincides with the projective space $\mathbb{P}_{1}(\mathbb{R})$. In other words, we have the following result.

#### Proposition 2

*There is a one*-*to*-*one correspondence between the light rays of the spin factor* $\mathbb{R}\oplus \mathbb{R}^{2}$ *and the rank one projections of the Jordan algebra* ${\mathcal{H}}(2,\mathbb{R})$.

#### Proof

The correspondence is given by

9 $$ (1+\mathbf{v})/2\longmapsto \frac{1}{2} \begin{pmatrix} 1+v_{1} & v_{2} \\ v_{2} & 1-v_{1}\end{pmatrix}, $$

where $\mathbf{v}=v_{1}e_{1}+v_{2}e_{2}$ is a unit vector of $\mathbb{R}^{2}$. □

We will see in the next section that this result has a meaningful interpretation: there is a one-to-one correspondence between the light rays of the spin factor $\mathbb{R}\oplus \mathbb{R}^{2}$ and the pure state density matrices of the algebra $\mathcal{H}(2,\mathbb{R})$.

The positive cone $\mathcal{C}$ of the Jordan algebra $\mathcal{A}$ is the set of its positive elements, namely

10 $$ \mathcal{C}=\{a\in \mathcal{A}, a> 0\} . $$

It can be shown that $\mathcal{C}$ is the interior of the positive domain of $\mathcal{A}$ defined as the set of squares of $\mathcal{A}$. The convex cone $\mathcal{C}$ is symmetric: it is regular, homogeneous, and self-dual [10]. The positive cone $\mathcal{H}^{+}(2,\mathbb{R})$ of the algebra $\mathcal{H}(2,\mathbb{R})$ is the set of positive-definite symmetric matrices.

## Quantum preliminaries

This section is devoted to describing the state space of the real analog of the usual complex qubit. The so-called rebit is a two-level real quantum system whose Hilbert’s space is $\mathbb{R}^{2}$.

### Recalls on state spaces

The positive cone $\mathcal{C}$ is the set of positive observables. A state of $\mathcal{A}$ is a linear functional

11 $$ \langle \cdot \rangle :\mathcal{A}\longrightarrow \mathbb{R} $$

that is nonnegative: $\langle a\rangle \geq 0$, $\forall a\geq 0$, and normalized: $\langle 1\rangle =1$. Given an element *a* of $\mathcal{A}$, we denote by $L(a)$ the endomorphism of $\mathcal{A}$ defined by $L(a)(b)=a\circ b$ and $\operatorname{Trace} (a)$ its trace, i.e., $\operatorname{Trace} (a):=\operatorname{Trace} (L(a))$. Since $\mathcal{A}$ is formally real, the pairing

12 $$ \langle a,b\rangle =\operatorname{Trace}\bigl(\mathrm{L}(a) (b) \bigr)=\operatorname{Trace}(a\circ b) $$

is a real-valued inner product and one can identify any state with a unique element *ρ* of $\mathcal{A}$ by setting

13 $$ \langle a\rangle =\operatorname{Trace}(\rho \circ a) , $$

where $\rho \geq 0$ and $\operatorname{Trace}(\rho )=1$. Such *ρ*, for $\mathcal{A}=\mathcal{H}(2,\mathbb{R})$, is a so-called state density matrix [8]. Formula (13) gives the expectation value of the observable *a* in the state with density matrix *ρ*.

Regarding Proposition 1, the positive state density matrices of the algebra $\mathcal{H}(2,\mathbb{R})$ are in one-to-one correspondence with the elements of the future lightcone

14 $$ \mathcal{L}^{+}=\bigl\{ a=(\alpha +\mathbf{v}), \alpha > 0, a\cdot a> 0\bigr\} $$

that are of the form $a=(1+\mathbf{v})/2$, with $\Vert {\mathbf{v}}\Vert \leq 1$. One way to qualify states is to introduce the Von Neumann entropy [8, 37]. It is given by

15 $$ S(\rho )=-\operatorname{Trace}(\rho \log \rho ) . $$

It appears that $S(\rho )=0$ if and only if *ρ* satisfies $\rho \circ \rho =\rho $. The zero entropy state density matrices characterize pure states that afford a maximum of information. Among the other state density matrices, one is of particular interest. It is given by $\rho _{0}=\mathrm{Id}_{2}/2$ or $\rho _{0}=(1+0)/2$ ($\mathrm{Id}_{2}$ is the identity matrix) and is characterized by

16 $$ \rho _{0}=\mathop{\operatorname{argmax}}_{\rho } S(\rho ) . $$

The mixed state with density matrix $\rho _{0}$ is called the state of maximum entropy, $S(\rho _{0})$ being equal to log2. It provides the minimum of information. Using (13), we have

17 $$ \langle a\rangle _{0}=\frac{\operatorname{Trace}(a)}{2} . $$

Now, given an observable *a*, *a* acts on the state $\langle \cdot \rangle _{0}$ by the formula

18 $$ a:\langle \cdot \rangle _{0}\longmapsto \langle a\circ \cdot \rangle = \langle \cdot \rangle _{0,a} . $$

Since for any state *ρ* the element 2*ρ* is an observable, we get

19 $$ \langle a\rangle _{0,2\rho }=\langle 2\rho \circ a\rangle _{0}=\operatorname{Trace}( \rho \circ a)=\langle a\rangle $$

for all observable *a*. This means that any state with density matrix *ρ* can be obtained from the state of maximal entropy with density matrix $\rho _{0}$ using the action of the observable 2*ρ*.

### The two-level real quantum system

The pure state density matrices of the algebra $\mathcal{A}=\mathcal{H}(2,\mathbb{R})$ are of the form

20 $$ \frac{1}{2} \begin{pmatrix} 1+v_{1} & v_{2} \\ v_{2} & 1-v_{1}\end{pmatrix} , $$

where $\mathbf{v}=v_{1}e_{1}+v_{2}e_{2}$ is a unit vector of $\mathbb{R}^{2}$. They are in one-to-one correspondence with the light rays of the 3-dimensional Minkowski spacetime, see Proposition 2.

A classical representation of quantum states is the Bloch body [38]. An element *ρ* of $\mathcal{H}(2,\mathbb{R})$ is a state density matrix if and only if it can be written as follows:

21 $$ \rho (v_{1},v_{2})=\frac{1}{2}( \mathrm{Id}_{2}+\mathbf{v}\cdot \sigma )=\frac{1}{2}( \mathrm{Id}_{2}+v_{1} \sigma _{1}+v_{2} \sigma _{2}) , $$

where $\sigma =(\sigma _{1},\sigma _{2})$ with

22 $$ \sigma _{1}= \begin{pmatrix} 1 & 0 \\ 0 & -1\end{pmatrix},\qquad \sigma _{2}= \begin{pmatrix} 0 & 1 \\ 1 & 0\end{pmatrix} , $$

and $\mathbf{v}=v_{1}e_{1}+v_{2}e_{2}$ is a vector of $\mathbb{R}^{2}$ with $\Vert {\mathbf{v}}\Vert \leq 1$. The matrices $\sigma _{1}$ and $\sigma _{2}$ are Pauli-like matrices. In the usual framework of quantum mechanics, that is, when the observable algebra is the algebra $\mathcal{H}(2,\mathbb{C})$ of 2 by 2 hermitian matrices with complex entries, the Bloch body is the unit Bloch ball in $\mathbb{R}^{3}$. It represents the states of the two-level quantum system of a spin-$\frac{1}{2}$ particle, also called a qubit. In the present context, the Bloch body is the unit disk of $\mathbb{R}^{2}$ associated with a rebit. We give now more details on this system using the classical Dirac notations [29], bra, ket, etc. Let us denote by $|u_{1}\rangle $, $|d_{1}\rangle $, $|u_{2}\rangle $, and $|d_{2}\rangle $ the four state vectors defined by

23 $$ |u_{1}\rangle = \begin{pmatrix} 1 \\ 0\end{pmatrix} ,\qquad |d_{1}\rangle = \begin{pmatrix} 0 \\ 1\end{pmatrix} ,\qquad |u_{2}\rangle =\frac{1}{\sqrt{2}} \begin{pmatrix} 1 \\ 1\end{pmatrix} ,\qquad |d_{2}\rangle =\frac{1}{\sqrt{2}} \begin{pmatrix} -1 \\ 1\end{pmatrix} . $$

We have

24 $$ \sigma _{1}= \vert u_{1}\rangle \langle u_{1} \vert - \vert d_{1}\rangle \langle d_{1} \vert ,\qquad \sigma _{2}= \vert u_{2} \rangle \langle u_{2} \vert - \vert d_{2}\rangle \langle d_{2} \vert . $$

The state vectors $|u_{1}\rangle $ and $|d_{1}\rangle $, resp. $|u_{2}\rangle $ and $|d_{2}\rangle $, are eigenstates of $\sigma _{1}$, resp. $\sigma _{2}$, with eigenvalues 1 and −1.

Using polar coordinates $v_{1}=r\cos \theta $, $v_{2}=r\sin \theta $, we can write $\rho (v_{1},v_{2})$ as follows:

25 $$\begin{aligned} \rho (r,\theta ) =&\frac{1}{2} \begin{pmatrix} 1+r\cos \theta & r\sin \theta \\ r\sin \theta & 1-r\cos \theta \end{pmatrix} \end{aligned}$$

26 $$\begin{aligned} =&\frac{1}{2} \bigl\{ (1+r\cos \theta ) \vert u_{1} \rangle \langle u_{1} \vert +(1-r \cos \theta ) \vert d_{1}\rangle \langle d_{1} \vert +(r\sin \theta ) \vert u_{2} \rangle \langle u_{2} \vert \end{aligned}$$

27 $$\begin{aligned} &{}-(r\sin \theta ) \vert d_{2}\rangle \langle d_{2} \vert \bigr\} . \end{aligned}$$

This gives, for instance,

28 $$\begin{aligned}& \rho (1,0)= \vert u_{1}\rangle \langle u_{1} \vert = \begin{pmatrix} 1 & 0 \\ 0& 0 \end{pmatrix} , \end{aligned}$$

29 $$\begin{aligned}& \rho (1,\pi )= \vert d_{1}\rangle \langle d_{1} \vert = \begin{pmatrix} 0 & 0 \\ 0& 1 \end{pmatrix} , \end{aligned}$$

30 $$\begin{aligned}& \rho (1,\pi /2)= \vert u_{2}\rangle \langle u_{2} \vert =\frac{1}{2} \begin{pmatrix} 1 & 1 \\ 1& 1 \end{pmatrix} , \end{aligned}$$

31 $$\begin{aligned}& \rho (1,3\pi /2)= \vert d_{2}\rangle \langle d_{2} \vert =\frac{1}{2} \begin{pmatrix} 1 & -1 \\ -1& 1 \end{pmatrix} . \end{aligned}$$

More generally

32 $$ \rho (1,\theta )= \bigl\vert (1,\theta )\bigr\rangle \bigl\langle (1,\theta ) \bigr\vert , $$

with

33 $$ \bigl|(1,\theta )\bigr\rangle =\cos (\theta /2) \vert u_{1}\rangle + \sin (\theta /2) \vert d_{1} \rangle . $$

This means that we can identify the pure state density matrices $\rho (1,\theta )$ with the state vectors $|(1,\theta )\rangle $ and also with the points of the unit disk boundary with coordinate *θ*. The state of maximal entropy, given by the state density matrix

34 $$ \rho _{0}=\frac{1}{2} \begin{pmatrix} 1 & 0 \\ 0 & 1\end{pmatrix} , $$

is the mixture

35 $$\begin{aligned} \rho _{0} =&\frac{1}{4} \vert u_{1}\rangle \langle u_{1} \vert +\frac{1}{4} \vert d_{1} \rangle \langle d_{1} \vert + \frac{1}{4} \vert u_{2}\rangle \langle u_{2} \vert +\frac{1 }{4} \vert d_{2} \rangle \langle d_{2} \vert \end{aligned}$$

36 $$\begin{aligned} =&\frac{1}{4}\rho (1,0)+\frac{1}{4}\rho (1,\pi )+ \frac{1}{4}\rho (1,\pi /2)+\frac{1 }{4}\rho (1,3\pi /2) , \end{aligned}$$

with equal probabilities. Using (25), we can write every state density matrix as follows:

37 $$ \rho (r,\theta )=\rho _{0}+\frac{r\cos \theta }{2} \bigl(\rho (1,0)- \rho (1,\pi ) \bigr)+\frac{r\sin \theta }{2} \bigl(\rho (1,\pi /2)- \rho (1,3\pi /2) \bigr) . $$

Such a state density matrix is given by the point of the unit disk with polar coordinates $(r,\theta )$. It is important to notice that the four state density matrices $\rho (1,0)$, $\rho (1,\pi )$, $\rho (1,\pi /2)$, and $\rho (1,3\pi /2)$ correspond to two pairs of state vectors $(|u_{1}\rangle , |d_{1}\rangle )$, $(|u_{2}\rangle , |d_{2}\rangle )$, the state vectors $|u_{i}\rangle $ and $|d_{i}\rangle $, for $i=1,2$, being linked by the “up and down” Pauli-like matrix $\sigma _{i}$.

### Remarks

In the usual framework, that is, when $\mathcal{A}$ is $\mathcal{H}(2,\mathbb{C})$, the three Pauli matrices are associated with the three directions of rotations in $\mathbb{R}^{3}$. In our case, there are only two Pauli-like matrices. The interpretation in terms of rotations ceases to be relevant since there is no space with a rotation group of dimension 2. This makes rebits somewhat strange. We explain in Sect. 7.1 that this real quantum system seems to be well adapted to provide a mathematical model of the opponency color mechanism of E. Hering.

The pure and mixed states play a crucial role in the measurements: “… that is, after the interaction with the apparatus, the system-plus-apparatus behaves like a mixture… It is in this sense, and in this sense alone, that a measurement is said to change a pure state into a mixture” [39] (see also the cited reference [40]). It seems actually that the problem of measurements in quantum mechanics was one of the main motivations of P. Jordan for the introduction of his new kind of algebras: “Observations not only disturb what has to be measured, they produce it… We ourselves produce the results of measurements” [39], p. 161, [41, 42].

## The Riemannian geometry of $\mathcal{C}$ and ${\mathcal{L}}^{+}$

Now, we give further information on the underlying geometry of the Jordan algebra $\mathcal{A}$ from both the points of view discussed above, that is, $\mathcal{A}$ as the algebra $\mathcal{H}(2,\mathbb{R})$ and $\mathcal{A}$ as the spin factor $\mathbb{R}\oplus \mathbb{R}^{2}$. We first recall how to endow the positive cone $\mathcal{C}$ of the algebra $\mathcal{H}(2,\mathbb{R})$ with a metric that makes $\mathcal{C}$ foliated by leaves isometric to the Poincaré disk. This is essentially the way followed by H.L. Resnikoff in [5] to obtain the geometric model $\mathcal{P}_{2}$. It appears that this geometric structure is not well adapted to our quantum viewpoint since it does not take into account the specific role of the density matrices. Instead, we propose to equip the positive cone $\mathcal{L}^{+}$ of the spin factor $\mathbb{R}\oplus \mathbb{R}^{2}$ with a metric that makes $\mathcal{L}^{+}$ foliated by leaves isometric to the Klein disk. This geometric structure is more appropriate to our approach since the state space considered before is naturally embedded in $\overline{\mathcal{L}^{+}}$ as a leaf, see (76).

### The Poincaré geometry of $\mathcal{C}$

We consider the level set

38 $$ \mathcal{C}_{1}=\bigl\{ X\in \mathcal{H}^{+}(2, \mathbb{R}), \operatorname{Det}(X)=1\bigr\} . $$

Every *X* in $\mathcal{C}_{1}$ can be written as follows:

39 $$ X= \begin{pmatrix} \alpha +v_{1} & v_{2} \\ v_{2} & \alpha -v_{1}\end{pmatrix} , $$

with $\mathbf{v}=v_{1}e_{1}+v_{2}e_{2}$ a vector of $\mathbb{R}^{2}$ satisfying $\alpha ^{2}-\Vert {\mathbf{v}}\Vert ^{2}=1$ and $\alpha >0$. Using the one-to-one correspondence

40 $$ X= \begin{pmatrix} \alpha +v_{1} & v_{2} \\ v_{2} & \alpha -v_{1}\end{pmatrix}\longmapsto (\alpha +\mathbf{v}) , $$

the level set $\mathcal{C}_{1}$ is sent to the level set

41 $$ \mathcal{L}_{1}=\bigl\{ a=(\alpha +\mathbf{v})\in \mathcal{L}^{+}, a\cdot a=1\bigr\} $$

of the future lightcone $\mathcal{L}^{+}$. It is well known that the projection

42 $$ \pi _{1}:\mathcal{L}_{1}\longrightarrow \{\alpha =0\} , $$

defined by

43 $$ \pi _{1}(\alpha +\mathbf{v})=(0+\mathbf{w}) , $$

with $\mathbf{w}=w_{1}e_{1}+w_{2}e_{2}$ and

44 $$ w_{i}=\frac{v_{i}}{1+\alpha } , $$

for $i=1,2$, is an isometry between the level set $\mathcal{L}_{1}$ and the Poincaré disk $\mathcal{D}$ [43]. Simple computations show that the matrix *X* can be written as follows:

45 $$ X= \begin{pmatrix} \frac{1+2w_{1}+(w_{1}^{2}+w_{2}^{2})}{1-(w_{1}^{2}+w_{2}^{2})} & \frac{2w_{2}}{1-(w_{1}^{2}+w_{2}^{2})} \\ \frac{2w_{2}}{1-(w_{1}^{2}+w_{2}^{2})} & \frac{1-2w_{1}+(w_{1}^{2}+w_{2}^{2}) }{1-(w_{1}^{2}+w_{2}^{2})}\end{pmatrix} $$

in the **w**-parametrization.

#### Proposition 3

*Let* *X* *be an element of* $\mathcal{C}_{1}$ *written under the form* (45), *we have*

46 $$ \frac{\operatorname{Trace} [(X^{-1}\,dX)^{2} ]}{2}=4 \biggl(\frac{(dw_{1})^{2}+(dw_{2})^{2} }{(1-(w_{1}^{2}+w_{2}^{2}))^{2}} \biggr)=ds^{2}_{\mathcal{D}} . $$

#### Proof

Cayley–Hamilton theorem implies the following equality, where *A* denotes a 2 by 2 matrix:

47 $$ \bigl(\operatorname{Trace}(A)\bigr)^{2}=\operatorname{Trace}\bigl( A^{2}\bigr)+2\operatorname{Det}(A) . $$

We apply this equality to the matrix $A=X^{-1}\,dX$. The matrix *X* can be written as follows:

48 $$ X= \biggl(\frac{1+ \vert z \vert ^{2}}{1- \vert z \vert ^{2}} \biggr)I_{2}+\frac{X_{1}}{1- \vert z \vert ^{2}} , $$

where

49 $$ X_{1}= \begin{pmatrix} 2w_{1} & 2w_{2} \\ 2w_{2} &-2w_{1} \end{pmatrix} $$

and $z=w_{1}+iw_{2}$. We have

50 $$ X^{-1}= \biggl(\frac{1+ \vert z \vert ^{2}}{1- \vert z \vert ^{2}} \biggr)I_{2}- \frac{X_{1}}{1- \vert z \vert ^{2}} $$

and

51 $$ dX=d \biggl(\frac{1+ \vert z \vert ^{2}}{1- \vert z \vert ^{2}} \biggr)I_{2}+\frac{dX_{1}}{1- \vert z \vert ^{2}}+ \frac{d( \vert z \vert ^{2}) }{(1- \vert z^{2} \vert )^{2}}X_{1} . $$

Consequently,

52 $$ X^{-1}\,dX=\frac{2\, d( \vert z \vert ^{2})}{(1- \vert z \vert ^{2})^{2}}I_{2} -\frac{d( \vert z \vert ^{2})}{(1- \vert z \vert ^{2})^{2}}X_{1}+ \frac{(1+ \vert z \vert ^{2})\,dX_{1} }{(1- \vert z \vert ^{2})^{2}}-\frac{X_{1}\,dX_{1}}{(1- \vert z \vert ^{2})^{2}} . $$

Since $\operatorname{Trace}(X_{1})=\operatorname{Trace}(dX_{1})=0$ and $\operatorname{Trace}(X_{1}\,dX_{1})=4d(|z|^{2})$, then

53 $$ \bigl[\operatorname{Trace}\bigl(X^{-1}\,dX\bigr) \bigr]^{2}=0 $$

and

54 $$ \operatorname{Trace} \bigl[\bigl(X^{-1}\,dX\bigr)^{2} \bigr]=-2\operatorname{Det}\bigl(X^{-1}\,dX\bigr)=-2 \operatorname{Det}(dX) . $$

We have also

55 $$\begin{aligned} dX =&\frac{d( \vert z \vert ^{2})}{(1- \vert z \vert ^{2})^{2}} \begin{pmatrix} 1+ \vert z \vert ^{2}+2w_{1} & 2w_{2} \\ 2w_{2} &1+ \vert z \vert ^{2}-2w_{1} \end{pmatrix} \end{aligned}$$

56 $$\begin{aligned} &{}+\frac{1}{(1- \vert z \vert ^{2})} \begin{pmatrix} d( \vert z \vert ^{2})+2\,dw_{1} & 2\,dw_{2} \\ 2\,dw_{2} &d( \vert z \vert ^{2})-2\,dw_{1} \end{pmatrix} . \end{aligned}$$

Simple computations lead to

57 $$ \operatorname{Det}(dX)=-4 \biggl(\frac{(dw_{1})^{2}+(dw_{2})^{2}}{(1- \vert z \vert ^{2})^{2}} \biggr) $$

and end the proof. □

This proposition means that $\mathcal{C}_{1}$ equipped with the normalized Rao–Siegel metric, i.e., $\operatorname{Trace} [(X^{-1}\,dX)^{2} ]/2$, is isometric to the Poincaré disk $\mathcal{D}$ of constant negative curvature equal to −1. Actually, $\mathcal{C}$ is foliated by the level sets of the determinant with leaves that are isometric to $\mathcal{D}$. This description, which is analogous to the one considered by H.L. Resnikoff in [5], does not take into account the specific role of the state density matrices of the algebra $\mathcal{H}(2,\mathbb{R})$.

### The Klein geometry of ${\mathcal{L}}^{+}$

Another classical result of hyperbolic geometry asserts that the projection

58 $$ \varpi _{1}:\mathcal{L}_{1}\longrightarrow \{\alpha =1 \} , $$

defined by

59 $$ \varpi _{1}(\alpha +\mathbf{v})=(1+x) , $$

with $x=x_{1}e_{1}+x_{2}e_{2}$ and

60 $$ x_{i}=\frac{v_{i}}{\alpha } , $$

for $i=1,2$, is an isometry between the level set $\mathcal{L}_{1}$ and the Klein disk $\mathcal{K}$, the Riemannian metric of which is given by

61 $$ ds^{2}_{\mathcal{K}}=\frac{(dx_{1})^{2}+(dx_{2})^{2}}{1-(x_{1}^{2}+x_{2}^{2})} +\frac{(x_{1}\,dx_{1}+x_{2}\,dx_{2})^{2} }{(1-(x_{1}^{2}+x_{2}^{2}))^{2}} , $$

see [43]. An isometry between $\mathcal{K}$ and $\mathcal{D}$ is defined by

62 $$\begin{aligned}& x_{i}=\frac{2w_{i}}{1+(w_{1}^{2}+w_{2}^{2})} , \end{aligned}$$

63 $$\begin{aligned}& w_{i}=\frac{x_{i}}{1+\sqrt{1-(x_{1}^{2}+x_{2}^{2})}} \end{aligned}$$

for $i=1,2$. In other words, we have the following commutative diagram of isometries: 64

Let us recall that the state density matrices of the quantum system we consider can be identified with the elements

65 $$ a=(1+\mathbf{v})/2 $$

of the spin factor $\mathbb{R}\oplus \mathbb{R}^{2}$ with $\Vert {\mathbf{v}}\Vert \leq 1$. Let us denote by

66 $$ \varpi _{1/2}:\mathcal{L}_{1/2}=\bigl\{ a=(\alpha + \mathbf{v})/2, \alpha >0, a \cdot a=1/4\bigr\} \longrightarrow \{\alpha =1/2\} $$

the projection given by

67 $$ \varpi _{1/2}\bigl((\alpha +\mathbf{v})/2\bigr)=(1+\mathbf{v}/\alpha )/2 . $$

We have

68 $$ \varpi _{1/2}\bigl((\alpha +\mathbf{v})/2\bigr)=\varpi _{1}(\alpha +\mathbf{v})/2 . $$

This means that the map

69 $$ \varphi :\mathcal{K}\longmapsto \mathcal{K}_{1/2} , $$

defined by

70 $$ \varphi (x_{1},x_{2})=(x_{1},x_{2})/2 , $$

is an isometry between $\mathcal{K}$ and

71 $$ \mathcal{K}_{1/2}=\bigl\{ x/2\in \mathbb{R}^{2}, \Vert x \Vert ^{2}< 1\bigr\} , $$

the Riemannian metric on the latter being given by

72 $$ ds^{2}_{\mathcal{K}_{1/2}}=\bigl(\varphi ^{-1} \bigr)^{*}\,ds^{2}_{\mathcal{K}}=\frac{(dx_{1})^{2}+(dx_{2})^{2} }{1/4-(x_{1}^{2}+x_{2}^{2})}+ \frac{(x_{1}\,dx_{1}+x_{2}\,dx_{2})^{2}}{(1/4-(x_{1}^{2}+x_{2}^{2}))^{2}} . $$

One can verify that $\mathcal{L}^{+}$ is foliated by the level sets $\alpha =\mathrm{constant}$ with leaves that are isometric to the Klein disk $\mathcal{K}$. This description is more appropriate than the above one to characterize perceived colors from real quantum states since the state space $\mathcal{S}$ is naturally embedded in $\overline{\mathcal{L}^{+}}$, see (76) below.

### Klein and Hilbert metrics

As an introduction to the discussions of Sects. 7.2 and 7.3, let us recall some basic facts about the geometry of the Klein disk. Contrary to the Poincaré disk, the geodesics of $\mathcal{K}$ are straight lines and more precisely the chords of the unit disk. An important feature of the Klein metric is that it coincides with the Hilbert metric defined as follows. Let *p* and *q* be two interior points of the disk, and let *r* and *s* be the two points of the boundary of the disk such that the segment $[r,s]$ contains the segment $[p,q]$. The Hilbert distance between *p* and *q* is defined by

73 $$ d_{H}(p,q)=\frac{1}{2}\log [r,p,q,s] , $$

where

74 $$ [r,p,q,s]=\frac{ \Vert q-r \Vert }{ \Vert p-r \Vert } \times \frac{ \Vert p-s \Vert }{ \Vert q-s \Vert } $$

is the cross-ratio of the four points *r*, *p*, *q*, and *s* [44] (in (74), $\Vert \cdot \Vert $ is the Euclidean norm).

## Perceived colors and chromatic states

We describe the space $\mathcal{P}$ of perceived colors under the only hypothesis that $\mathcal{P}$ can be described from the state space of a quantum system characterized by the Jordan algebra $\mathcal{H}(2,\mathbb{R})$. As explained before, we exploit the fact that $\mathcal{H}(2,\mathbb{R})$ is isomorphic to the spin factor $\mathbb{R}\oplus \mathbb{R}^{2}$. Let us recall that this description does not involve any reference to physical colors or to an observer.

### Perceived colors as quantum measurements

The state space $\mathcal{S}$ is the unit disk embedded in the space of state density matrices by

75 $$ s=(v_{1},v_{2})\longmapsto \rho (v_{1},v_{2})= \frac{1}{2} \begin{pmatrix} 1+v_{1} & v_{2} \\ v_{2} & 1-v_{1}\end{pmatrix} $$

and in the Klein disk $\mathcal{K}_{1/2}$ of the closure $\overline{\mathcal{L}^{+}}$ of the future lightcone $\mathcal{L}^{+}$ by

76 $$ s=(v_{1},v_{2})\longmapsto \frac{1}{2}(1+ \mathbf{v})=1/2+(v_{1}/2,v_{2}/2) . $$

In order to describe perceived colors, i.e., measured colors, it is necessary to characterize all the possible measurements that can be performed on the states. We adopt here the viewpoint of the generalized probability theory [45]. The reader may refer for instance to [46, 47], and [48] for more information on related topics.

We denote by $\mathcal{C}(\mathcal{S})$ the state cone defined by

77 $$ \mathcal{C}(\mathcal{S})=\left \{ \alpha \begin{pmatrix} v_{1} \\ v_{2} \\ 1\end{pmatrix}, \alpha \geq 0, s=(v_{1},v_{2}) \in \mathcal{S}\right \} . $$

This cone is self-dual, that is,

78 $$ \mathcal{C}(\mathcal{S})=\mathcal{C}^{*}(\mathcal{S})=\bigl\{ a\in \mathcal{A}, \forall b\in \mathcal{C}(\mathcal{S}), \langle a,b\rangle \geq 0\bigr\} , $$

where $\langle \cdot ,\cdot \rangle $ denotes the inner product of $\mathcal{A}$ given by (12). By definition, an effect is an element *e* of $\mathcal{C}^{*}(\mathcal{S})$ such that $e(s)\leq 1$ for all *s* in $\mathcal{S}$. Such an effect *e* can be seen as an affine function $e:\mathcal{S}\longrightarrow [0,1]$ with $0\leq e(s)\leq 1$ for all *s*. It is the most general way of assigning a probability to all states. Effects correspond to positive operator-valued measures. They also correspond to nonnegative symmetric matrices. In fact, the cone $\mathcal{C}(\mathcal{S})$ is the positive domain of the algebra $\mathcal{A}$. Considering $\mathcal{A}$ as the algebra $\mathcal{H}(2,\mathbb{R})$, this means that an effect *e* is a symmetric matrix such that $\langle e,f\rangle =\operatorname{Trace}(ef)\geq 0$ for all nonnegative symmetric matrix *f*. In order to verify that *e* is nonnegative, let us suppose that this is not the case and thus that one of the eigenvalues of *e* is negative, the corresponding eigenvector being denoted by **w**. One can then check that the trace of the product $e\mathbf{w}{\mathbf{w}}^{t}$ is negative. This gives a contradiction since the matrix $f=\mathbf{w}{\mathbf{w}}^{t}$ is symmetric and nonnegative.

In the present settings, every effect is given by a vector $e=(a_{1},a_{2},a_{3})$ such that

79 $$ 0\leq e\cdot \begin{pmatrix} v_{1} \\ v_{2} \\ 1\end{pmatrix}\leq 1 $$

for all $s=(v_{1},v_{2})$ in $\mathcal{S}$. The measurement effect associated with *e* is the operator

80 $$ E=a_{3}\,\mathrm{Id}_{2}+a_{1}\sigma _{1}+a_{2}\sigma _{2} $$

that must satisfy $0\leq E\leq \mathrm{Id}_{2}$. This last condition implies that $0\leq a_{3}\leq 1$, with $a_{1}^{2}+a_{2}^{2}\leq a_{3}^{2}$ and $a_{1}^{2}+a_{2}^{2}\leq (1-a_{3})^{2}$. We denote by $\mathcal{E}(\mathcal{S})$ the effect space of $\mathcal{S}$, that is, the set of all effects on $\mathcal{S}$. As explained in Sect. 7.1, this space appears to coincide with the “double cone” depicted in Fig. 4.11 of [17], p. 123. Note that the so-called unit effect, $e_{1}=(0,0,1)$, satisfies $e_{1}(s)=1$ for all *s* in $\mathcal{S}$.

### Colorimetric definitions

A perceived color $c=(a_{1},a_{2},a_{3})$ is by definition an effect on $\mathcal{S}$, that is, an element of the effect space $\mathcal{E}(\mathcal{S})$. Since $\mathcal{C}(\mathcal{S})=\mathcal{C}^{*}(\mathcal{S})$, a perceived color *c* is an element of the state cone of $\mathcal{S}$, this one being the closure $\overline{\mathcal{L}^{+}}$ of the future lightcone $\mathcal{L}^{+}$. The element $c/(2 a_{3})=(a_{1}/2a_{3},a_{2}/2a_{3},1/2)$, $a_{3}\neq 0$, belongs to the Klein disk $\mathcal{K}_{1/2}$ of $\overline{\mathcal{L}^{+}}$. This suggests to define the colorimetric attributes of *c* as follows:

- The real $a_{3}$, with $0\leq a_{3}\leq 1$, is the magnitude of the perceived color *c*.
- The element $s_{c}=(a_{1}/a_{3},a_{2}/a_{3})\in \mathcal{S}$ is the chromatic state of *c*.
- A perceived color with a unit chromatic state is a pure perceived color.
- The saturation of a perceived color *c* is given by the Von Neumann entropy of its chromatic state.
- A perceived color whose chromatic state is the state of maximal entropy is achromatic.

Given a state $(v_{1},v_{2})\in \mathcal{S}$, the perceived colors which have this state as chromatic state form the intersection

81 $$ c_{s}=\mathcal{E}(\mathcal{S})\cap \left \{ \begin{pmatrix} a_{3}v_{1} \\ a_{3}v_{2} \\ a_{3}\end{pmatrix}, 0\leq a_{3}\leq 1\right \} . $$

The maximum value of a perceived color $c=(a_{1},a_{2},a_{3})$ is

82 $$ \begin{pmatrix} a_{1} \\ a_{2} \\ a_{3}\end{pmatrix}\cdot \begin{pmatrix} a_{1}/a_{3} \\ a_{2}/a_{3} \\ 1\end{pmatrix}=\frac{a_{1}^{2}+a_{2}^{2}}{a_{3}}+a_{3}=a_{3} \bigl(1+r^{2}\bigr) , $$

with $r^{2}=(a_{1}^{2}+a_{2}^{2})/a_{3}^{2}$. We must have

83 $$ 0\leq r^{2}\leq \frac{1-a_{3}}{a_{3}}\leq 1 . $$

If $0< a_{3}<1/2$, the measure of a perceived color $c=(a_{1},a_{2},a_{3})$ on its chromatic state $(a_{1}/a_{3},a_{2}/a_{3})$ gives the probability $a_{3}(1+r^{2})$, this one being well defined for all $0< r\leq 1$. In particular, pure perceived colors are measured with the maximum probability $2a_{3}$. In this case, the magnitude is not high enough to allow measurements with probability 1, and the perceived colors are under-estimated.

If $a_{3}=1/2$, the measure of a perceived color $c=(a_{1},a_{2},1/2)$ on its chromatic state $(2a_{1},2a_{2})$ gives the probability $(1+r^{2})/2$. This probability is well defined for all $0< r\leq 1$. It is maximal, equal to 1, if and only if *c* is a pure perceived color. In this case, the perceived colors are ideally-estimated.

If $1/2< a_{3}<1$, the measure of a perceived color $c=(a_{1},a_{2},a_{3})$ on its chromatic state $(a_{1}/a_{3},a_{2}/a_{3})$ gives the probability $a_{3}(1+r^{2})$. This probability is well defined if and only if equation (83) is satisfied. In particular, pure perceived colors cannot be measured on their chromatic states. For instance, if $a_{3}=2/3$, then *r* should be less than or equal to $\sqrt{2}/2$ and perceived colors with chromatic states of norm equal to $\sqrt{2}/2$ are measured with probability 1. In this case, the perceived colors are over-estimated.

An achromatic perceived color $c=(0,0,a_{3})$ measured on a chromatic state gives the probability $a_{3}$ and this independently of the considered chromatic state. Such a perceived color does not take into account chromaticity. The unit perceived color $c=e_{1}$ is the saturated achromatic perceived color.

## Group actions and homogeneity

As already mentioned, Resnikoff’s work is based on the fact that there should exist a linear group acting transitively on the space of perceived colors [5, 9]. The elements of this group are supposed to be background illuminant changes. Up to now, we have not taken into account such an action to obtain the description that we propose for the space of perceived colors. This section is mainly devoted to showing that one can characterize illumination changes by Lorentz boost maps starting from the quantum dynamics described above. We will see in Sect. 7.2 how to relate our results with those of H. Yilmaz [14, 15].

### Recalls on the special Lorentz group

Let us first recall that the special Lorentz group $\operatorname{SO}^{+}(1,2)$ is the identity component of the group $O(1,2)$, this latter being the matrix Lie group that preserves the quadratic form

84 $$ \bigl\Vert (\alpha +\mathbf{v}) \bigr\Vert _{\mathcal{M}}=\alpha ^{2}- \Vert {\mathbf{v}} \Vert ^{2}, $$

where $(\alpha +\mathbf{v})$ belongs to the spin factor $\mathbb{R}\oplus \mathbb{R}^{2}$. The fact that $\operatorname{SO}^{+}(1,2)$ acts linearly on $\mathcal{L}^{+}$ means that it acts projectively on the set of lines of $\mathcal{L}^{+}$ and consequently on the points of the Klein disk $\mathcal{K}_{1/2}$ [49]. Moreover, this projective action gives the isometries of $\mathcal{K}_{1/2}$.

The subgroup of $\operatorname{SO}^{+}(1,2)$ that fixes $(1+0)$ may be identified with the group of rotations $\operatorname{SO}(2)$, and in fact every element *g* of $\operatorname{SO}^{+}(1,2)$ can be decomposed in a unique way as follows [50]:

85 $$ g=b_{\zeta }r_{\xi } , $$

where $b_{\zeta }$ is a boost map and $r_{\xi }$ is a proper rotation. More precisely, if we consider the coordinates $(\alpha , v_{1}, v_{2})$ in $\mathcal{L}^{+}$, the matrix associated with $b_{\zeta }$ is given by

86 $$ M(b_{\zeta })= \begin{pmatrix} \cosh (\zeta _{0})&\zeta _{x}\sinh (\zeta _{0}) & \zeta _{y}\sinh ( \zeta _{0}) \\ \zeta _{x}\sinh (\zeta _{0}) & 1+\zeta _{x}^{2}(\cosh (\zeta _{0})-1) & \zeta _{x}\zeta _{y}(\cosh (\zeta _{0})-1) \\ \zeta _{y}\sinh (\zeta _{0}) & \zeta _{x}\zeta _{y}(\cosh (\zeta _{0})-1)& 1+\zeta _{y}^{2}(\cosh (\zeta _{0})-1) \end{pmatrix} , $$

where $(\zeta _{x},\zeta _{y})$ is a unit vector of $\mathbb{R}^{2}$ and $\zeta _{0}$ is the rapidity of the boost. It should be noted that the set of boosts is not a subgroup of the special Lorentz group. The matrix associated with $r_{\xi }$ is given by

87 $$ M(r_{\xi })= \begin{pmatrix} 1 & 0 & 0 \\ 0 & \cos \xi & -\sin \xi \\ 0 & \sin \xi & \cos \xi \end{pmatrix} . $$

In order to illustrate the projective action of the boost $b_{\zeta }$ on the Klein disk $\mathcal{K}_{1/2}$, let us consider a simple example (quite more complicated computations give similar results in the general case). We choose $\zeta _{x}=1$, $\zeta _{y}=0$, and denote $\overline{\zeta }=\tanh (\zeta _{0})$. The image $(\alpha ,w_{1},w_{2})$ of a vector $(1/2,\cos \theta /2,\sin \theta /2)$ is given by

88 $$ \textstyle\begin{cases} 2\alpha = \cosh (\zeta _{0})+\sinh (\zeta _{0})\cos \theta, \\ 2w_{1} = \sinh (\zeta _{0})+\cosh (\zeta _{0})\cos \theta, \\ 2w_{2}=\sin \theta . \end{cases} $$

This means that the image of the boundary point $(\cos \theta /2,\sin \theta /2)$ is the boundary point $(v_{1},v_{2})$ with

89 $$ \textstyle\begin{cases} 2v_{1}= \frac{\overline{\zeta }+\cos \theta }{1+ \overline{\zeta }\cos \theta }, \\ 2v_{2}= \frac{(1-\overline{\zeta }^{2})^{1/2}\sin \theta }{1+ \overline{\zeta }\cos \theta } . \end{cases} $$

One may notice that the map sending the point $(\cos \theta /2,\sin \theta /2)$ to the point $(v_{1},v_{2})$ is an element of the group $\operatorname{PSL}(2,\mathbb{R})$. Transformation (89) will be used in Sect. 7.2 to interpret Yilmaz third experiment as a colorimetric analog of the relativistic aberration effect.

### Pure states and one parameter subgroups of Lorentz boosts

The fact that boost maps act on pure states with $\operatorname{PSL}(2,\mathbb{R})$ transformations is not surprising in view on the following result.^2^

#### Proposition 4

*Every pure state generates a one*-*parameter subgroup of boosts*.

#### Proof

As seen before, the state density matrix of a pure state is given by

90 $$ \rho (v_{1},v_{2})=\frac{1}{2}( \mathrm{Id}_{2}+\mathbf{v}\cdot \sigma ) , $$

where $\mathbf{v}=(v_{1},v_{2})$ is a unit vector and $\sigma =(\sigma _{1},\sigma _{2})$. The matrices $\sigma _{1}$ and $\sigma _{2}$ are symmetric traceless matrices that are usually chosen to be the two first generators of the Lie algebra $\operatorname{sl}(2,\mathbb{R})$ of the group $\operatorname{SL}(2,\mathbb{R})$. Note that they do not generate a sub-Lie algebra of $\operatorname{sl}(2,\mathbb{R})$. The matrix

91 $$ A(\rho ,\zeta _{0})=\exp \biggl(\zeta _{0} \frac{\mathbf{v}\cdot \sigma }{2} \biggr) $$

is a symmetric element of $\operatorname{PSL}(2,\mathbb{R})$, $\zeta _{0}$ being a real parameter. Let us recall that the $\operatorname{PSL}(2,\mathbb{R})$ action on $\mathcal{H}(2,\mathbb{R})$ is defined by

92 $$ X\longmapsto AXA^{t} . $$

We have clearly $\operatorname{Det}(AXA^{t})=\operatorname{Det}(X)$. Since $\sigma _{1}$ and $\sigma _{2}$ are elements of $\mathcal{H}(2,\mathbb{R})$, we can consider the matrices given by

93 $$ \sigma _{i}\longmapsto A(\rho ,\zeta _{0})\sigma _{i}A(\rho ,\zeta _{0}) $$

for $i=0,1,2$, with $\sigma _{0}=\mathrm{Id}_{2}$. It can be shown that the $3\times 3$ matrix with coefficients

94 $$ M(\rho ,\zeta _{0})_{ij}=\frac{1}{2}{ \operatorname{Trace}} \bigl(\sigma _{i}A( \rho ,\zeta _{0}) \sigma _{j}A(\rho ,\zeta _{0}) \bigr) $$

is a boost $b_{\zeta }$ with $\zeta =\tanh (\zeta _{0})(v_{1},v_{2})$. Let us verify it on a simple example where $v_{1}=1$ and $v_{2}=0$. In this case,

95 $$ A(\rho ,\zeta _{0})=\exp \biggl(\zeta _{0} \frac{v_{1}\sigma _{1}}{2} \biggr)=\exp \biggl(\zeta _{0} \frac{\sigma _{1}}{2} \biggr)= \begin{pmatrix} e^{\zeta _{0}/2} & 0 \\ 0 & e^{-\zeta _{0}/2}\end{pmatrix} . $$

We only need to compute the coefficient $B(\sigma ,\zeta _{0})_{i,j}$ for $i\leq j$. We have

96 $$ \textstyle\begin{cases} M(\sigma ,\zeta _{0})_{00}=\frac{1}{2}\operatorname{Trace} (A^{2}(\rho , \zeta _{0}) )=\cosh (\zeta _{0}), \\ M(\sigma ,\zeta _{0})_{01}=\frac{1}{2}\operatorname{Trace} (A(\rho ,\zeta _{0}) \sigma _{1}A(\rho ,\zeta _{0}) )=\sinh (\zeta _{0}), \\ M(\sigma ,\zeta _{0})_{02}=\frac{1}{2}\operatorname{Trace} (A(\rho ,\zeta _{0}) \sigma _{2}A(\rho ,\zeta _{0}) )=0, \\ M(\sigma ,\zeta _{0})_{11}=\frac{1}{2}\operatorname{Trace} (\sigma _{1} A( \rho ,\zeta _{0})\sigma _{1}A(\rho ,\zeta _{0}) )=\cosh ( \zeta _{0}), \\ M(\sigma ,\zeta _{0})_{12}=\frac{1}{2}\operatorname{Trace} ( \sigma _{1}A( \rho ,\zeta _{0})\sigma _{2}A(\rho ,\zeta _{0}) )=0, \\ M(\sigma ,\zeta _{0})_{22}=\frac{1}{2}\operatorname{Trace} ( \sigma _{2}A( \rho ,\zeta _{0})\sigma _{2}A(\rho ,\zeta _{0}) )=1 . \end{cases} $$

This means that $M(\rho ,\zeta _{0})=M(b_{\zeta })$ with $\zeta =\tanh (\zeta _{0})(1,0)$, see equation (86). □

One can easily verify that the image of the vector $(1/2,0,0)$ of $\mathcal{L}^{+}$ by the boost $b_{\zeta }=\tanh (\zeta _{0})(1,0)$ is the vector $(\cosh (\zeta _{0})/2,\sinh (\zeta _{0})/2,0)$. In consequence, the state of maximal entropy $\rho _{0}=(0,0)$ is sent to the state $(\tanh (\zeta _{0})/2,0)$. This extends to general boosts.

We can summarize these computations in the following way. As before, we consider the state space $\mathcal{S}$ as the Klein disk $\mathcal{K}_{1/2}$ of the closure $\overline{\mathcal{L}^{+}}$ of the future lightcone $\mathcal{L}^{+}$ by using the map

97 $$ s=(v_{1},v_{2})\longmapsto \frac{1}{2}(1+ \mathbf{v})=1/2+(v_{1}/2,v_{2}/2) . $$

Every pure state *ρ* generates a one-parameter subgroup of boosts, the parameter $\zeta _{0}$ being the rapidity. Actually, every boost can be obtained in this way. Boost maps act on the Klein disk $\mathcal{K}_{1/2}$ by isometries. If we consider $\mathcal{S}$ as embedded in the space of state density matrices by

98 $$ s=(v_{1},v_{2})\longmapsto \rho (v_{1},v_{2})= \frac{1}{2} \begin{pmatrix} 1+v_{1} & v_{2} \\ v_{2} & 1-v_{1}\end{pmatrix} , $$

the one-parameter subgroup of boosts is obtained by considering the action of $\operatorname{PSL}(2,\mathbb{R})$ on $\mathcal{H}(2,\mathbb{R})$. It is important to notice that we use only the matrices $\sigma _{0}$, $\sigma _{1}$, and $\sigma _{2}$, i.e., only information from $\mathcal{S}$. Since every state can be obtained from the state of maximal entropy, boosts, or equivalently pure states, act transitively on *S*. However, one has to pay attention to the fact that boost maps do not form a subgroup of the special Lorentz group, which is reflected by the fact that $\sigma _{1}$ and $\sigma _{2}$ do not form a sub-Lie algebra of the Lie algebra $\operatorname{sl}(2,\mathbb{R})$.

### About homogeneity

This point of view is quite different from the approach adopted in [5]. As mentioned in the introduction and explained in [9], one of the key arguments of H.L. Resnikoff is the existence of a transitive action of the group denoted $\operatorname{GL}(\mathcal{P})$ on the space $\mathcal{P}$ of perceived colors. This group is supposed to be composed of all linear changes of background illumination. In what precedes, we make use of the action of $\operatorname{PSL}(2,\mathbb{R})$ on $\mathcal{H}(2,\mathbb{R})$, see (92). But the matrices $A(\rho ,\zeta _{0})$ of $\operatorname{PSL}(2,\mathbb{R})$ that are used are also symmetric, due to the fact that $\sigma _{1}$ and $\sigma _{2}$ are symmetric. Actually, the action (92) can be also viewed as the action

99 $$ X\longmapsto AXA $$

of the Jordan algebra $\mathcal{H}(2,\mathbb{R})$ on itself. This is precisely the action

100 $$ Q(A):X\longmapsto \bigl(2L(A)^{2}-L\bigl(A^{2}\bigr) \bigr)X $$

of the quadratic representation of *A* on *X* [10]. But once again, the matrices *X* that we consider are $\sigma _{0}$, $\sigma _{1}$, and $\sigma _{2}$. The matrices $\sigma _{1}$ and $\sigma _{2}$ are not elements of the positive cone $\mathcal{H}^{+}(2,\mathbb{R})$. It appears in consequence that the homogeneity of $\mathcal{H}^{+}(2,\mathbb{R})$ is not so important in our approach. Instead of postulating the existence of a group of linear changes of background illumination, we have shown that the quantum description that we propose naturally leads to considering boost maps as illumination changes. These illumination changes are isometries of the Klein disk $\mathcal{K}_{1/2}$.

## Consequences and perspectives

We discuss now some consequences and perspectives of our results regarding color perception.

### Neural coding of colors and Hering’s rebit

D.H. Krantz describes in [51] Hering’s color opponency mechanism [52] as follows: “E. Hering noted that colors can be classified as reddish or greenish or neither, but that redness and greenness are not simultaneously attributes of a color. If we add increasing amounts of a green light to a reddish light, the redness of the mixture decreases, disappears, and gives way to greenness. At the point where redness is gone and greenness is not yet present, the color may be yellowish, bluish, or achromatic. We speak of a partial chromatic equilibrium, with respect to red/green… Similarly, yellow and blue are identified as opponent hues…”

Let us rename $|g\rangle =|u_{1}\rangle $, $|r\rangle =|d_{1}\rangle $, $|b\rangle =|u_{2}\rangle $, and $|y\rangle =|d_{2}\rangle $ as the four state vectors characterizing the rebit. The opponency mechanism is given by the two matrices $\sigma _{1}$ and $\sigma _{2}$. More precisely, the state vector

101 $$ \bigl|(1,\theta )\bigr\rangle =\cos (\theta /2) \vert g\rangle +\sin (\theta /2) \vert r \rangle $$

satisfies

102 $$ \bigl\langle (1,\theta ) \bigr\vert \sigma _{1} \bigl\vert (1,\theta ) \bigr\rangle =\cos \theta , \qquad \bigl\langle (1,\theta ) \bigr\vert \sigma _{2} \bigl\vert (1,\theta )\bigr\rangle =\sin \theta . $$

This means that if $\cos \theta > 0$, then the pure chromatic state $s(\theta )$ of the Bloch disk with coordinate *θ* is greenish, and if $\cos \theta < 0$, then $s(\theta )$ is reddish. For $\theta =\pi /2$, or $\theta =3\pi /2$, $s(\theta )$ is achromatic in the opposition green/red. In the same way, if sin*θ* is positive, then $s(\theta )$ is bluish, and if sin*θ* is negative, then $s(\theta )$ is yellowish. For $\theta =0$, or $\theta =\pi $, $s(\theta )$ is achromatic in the blue/yellow opposition. The phenomenon “that redness and greenness are not simultaneously attributes of a color”, for instance, is a trivial consequence of the fact that $\langle (1,\theta )|\sigma _{1}|(1,\theta )\rangle $ cannot be simultaneously positive and negative.

The mathematical description of the opponency that we propose seems to be relevant regarding the physiological mechanisms of the neural coding of colors [16] and [17]. These mechanisms involve both three separate receptor types (the L, M, and S cones) and spectrally opponent and nonopponent interactions. These latter, which take place at a higher level in the processing pipeline, result essentially from the activity rates of ganglion and lateral geniculate nucleus cells [53, 54]. Roughly speaking, color information is obtained by detecting and magnifying the differences between the various receptor type outputs.

Ganglion cells take their inputs from the bipolar and amacrine cells and relay the information to the lateral geniculate nucleus through ganglion axons. Most of the ganglion cells are on-center and off-surround, which means that they are activated if light falls in the center of their receptive fields and inhibited if light falls in the surround of their receptive fields. There exist also off-center and on-surround ganglion cells. One distinguishes two types of spectrally opponent interactions. The first one is given by the activity rate of midget ganglion cells located in the fovea. These cells fire when the difference of the spectral sensitivities of the L and M cones is greatest. This mechanism produces the L-M and M-L spectral opposition [53]. The second type is given by the activity rate of bistratified ganglion cells [54]. These cells fire when the difference between the spectral sensitivity of the S cone and both the spectral sensitivities of the L and M cones is greatest. This second mechanism produces the S-(L+M) and (L+M)-S spectral opposition. Besides the spectrally opponent interactions, there exists one type of spectrally nonopponent interaction given by the activity rate of parasol ganglion cells. These cells carry essentially the L+M and -(L+M) information.

As summarized in [17], the two main types of neural interactions seen in the precortical visual system are thus due to four spectrally opponent cells,^3^ R-G, G-R, B-Y, and Y-B, and to two spectrally nonopponent cells Bl and Wh. The hue of a perceived color is determined by the activity rates among the four spectrally opponent cell types, the lightness by the two activity rates of the spectrally nonopponent cells, and the saturation by the relative rates of the opponent and nonopponent cells. This description is clearly coherent with our results. As already mentioned, the “double cone” depicted in [17], Fig. 4.11, p. 123, is nothing else than the effect space of the real quantum system of Sect. 3.2. This justifies the terminology *Hering’s rebit*. This also shows that rebits, with only two opposition directions, can be relevant to model nonphysical phenomena related to perception.

It appears in consequence that the quantum model that we propose allows to recover axiomatically, starting from the sole trichromacy axiom, that a chromatic pure state, that is, a hue, is given by a pair of splittings similar to the two spin up and down inversions of a rebit. Following L.E.J. Brouwer, “Newton’s theory of color analyzed light rays in their medium, but Goethe and Schopenhauer, more sensitive to the truth, considered color to be the polar splitting by the human eye” [55] (see also [56] and [57]).

### Yilmaz’s relativity of color perception from the trichromacy axiom

Yilmaz contributions [14] and [15] are devoted to deriving colorimetric analogs of the relativistic Lorentz transformations from three basic experiments. The first experiment is supposed to show that color perception is a relativistic phenomenon; the second one to show that there exists a limiting saturation invariant under illumination changes; and the third one to show that there exists a colorimetric analog of the relativistic aberration effect [58]. These experiments involve observers located in two different rooms and who perform color matching according to illuminant changes. In particular, the interpretation of the third experiment is crucial for the derivation of the transformations since it avoids introducing a perceptually invariant quadratic form whose existence is very difficult to justify experimentally.^4^

Our objective here is to explain how to recover the result and the interpretation of this third experiment with the only use of the trichromacy axiom. The reader will find a more complete and detailed exposition in the forthcoming paper [59]. We have shown in Sect. 6 how to obtain the expression of the illuminant changes as Lorentz boost maps from the trichromacy axiom. We have also described the projective action of these transformations on the Klein disk $\mathcal{K}_{1/2}$ in the particular case that interests us here, see (89).

Under transformation (89), the image of the point $\overline{R}=(1/2,0)$ is the point $\overline{R}'=\overline{R}=(1/2,0)$ (we use the notations of [15]). So, this point remains unchanged. The image of the point $\overline{Y}=(0,1/2)$ is the point $\overline{Y}'=(\overline{\zeta }/2,(1-\overline{\zeta }^{2})^{1/2}/2)$ and the point *Y̅* has moved on the boundary, the angle *ϕ*, the one reported in [15], p. 12, being given by $\sin \phi =\overline{\zeta }$. When the rapidity $\zeta _{0}$ increases, *ζ̅* approaches 1 and the point $(\overline{\zeta },(1-\overline{\zeta }^{2})^{1/2})/2$ approaches the point $(1,0)/2$. At the limit $\overline{\zeta }=1$, every point $(\cos \theta ,\sin \theta )/2$ is sent to the point $(1,0)/2$, except the point $(-1,0)/2$. This means that every pure chromatic state, except the green pure chromatic state, can be transformed to a pure chromatic state arbitrarily close to the red pure chromatic state under the Lorentz boost if the rapidity $\zeta _{0}$ is sufficiently great. To explain the results of Yilmaz third experiment, note that $v_{1}$ in (89) is the cosine of the angle of the ray from the achromatic state to the image of the chromatic state $(\cos \theta ,\sin \theta )/2$ viewed under the initial illuminant *I*, whereas

103 $$ \overline{v}_{1}=\frac{-\overline{\zeta }+\cos \theta }{1-\overline{\zeta }\cos \theta } $$

is the cosine of the angle of the ray from the achromatic state to the image of the chromatic state $(\cos \theta ,\sin \theta )/2$ viewed under the illuminant $I'$. In consequence, under the illuminant $I'$, the expected yellow chromatic state given by $\theta =\pi /2$ is in fact the greenish chromatic state given by $\cos \theta =-\tanh (\zeta _{0})$.

We have already remarked in Sect. 4.3 that the hyperbolic Klein metric on $\mathcal{K}_{1/2}$ is given by the Hilbert metric. The relativistic viewpoint allows to better understand the relevance of this latter. One can first show that chromatic vectors satisfy a colorimetric analog of the Einstein–Poincaré addition law. More precisely, given to perceived colors *c* and *d* with chromatic vectors $\mathbf{v}_{c}=(v_{c},0)$ and $\mathbf{v}_{d}=(v_{d},0)$, the chromatic vector $\mathbf{v}^{c}_{d}=(v^{c}_{d},0)$ that describes the perceived color *c* with respect to *d* satisfies [59]

104 $$ {\mathbf{v}}_{c}=\frac{\mathbf{v}^{c}_{d}+\mathbf{v}_{d}}{1+4{\mathbf{v}}^{c}_{d}{ \mathbf{v}}_{d}} . $$

Then, this addition law can be related to an invariance property of the Hilbert metric. It is proven in [59] that

105 $$ d_{H}\bigl(\mathbf{0},\mathbf{v}^{c}_{d} \bigr)=d_{H}(\mathbf{v}_{d},\mathbf{v}_{c}) \quad \iff\quad { \mathbf{v}}_{c}=\frac{\mathbf{v}^{c}_{d}+\mathbf{v}_{d}}{1+4{\mathbf{v}}^{c}_{d}{ \mathbf{v}}_{d}} . $$

This last equivalence expresses the constancy of the Hilbert metric regarding illumination changes.

### On MacAdam ellipses and Hilbert metric

Hilbert’s metric is in fact defined on every convex set *Ω* and is always a Finsler metric^5^ [60, 61], whose asymmetric norm is given by

106 $$ \Vert {\mathbf{v}} \Vert _{p}=\frac{1}{2} \biggl( \frac{1}{ \Vert p-p^{-} \Vert }+\frac{1 }{ \Vert p-p^{+} \Vert } \biggr) \Vert {\mathbf{v}} \Vert , $$

where $p\in \varOmega $ and $p^{\pm }$ are the intersection points with the boundary *∂Ω* of the oriented line in *Ω* defined by the vector **v** with Euclidean norm $\Vert {\mathbf{v}}\Vert $ based at the point *p*. It is well known that this Finsler metric is Riemannian if and only if the boundary *∂Ω* is an ellipse.

The perceived color space that we have described is an ideal space that does not involve specific characteristics of a human observer. It is natural to envisage to characterize every human observer’s capability regarding color perception by a convex subset *Ω* of the state space $\mathcal{S}$, or equivalently of the Klein disk $\mathcal{K}_{1/2}$, endowed with the Finsler metric given by the Hilbert distance. This convex subset *Ω* is in some sense the restriction of the ideal chromatic state space due to the limitation of the observer perception. Work in progress is devoted to identifying, for each observer, the convex subset *Ω* by comparing the balls of the Finsler metric with the MacAdam ellipses drawn by the observer [62], which seem very similar.

Let us also mention that the problem of discernibility of perceived colors is reminiscent of the problem of distinguishability of quantum states [63, 64].

### Contexts and open quantum systems

It is important to notice that our study does not take into account so-called contextual effects, e.g., spatial context effects, that are involved in various well-known color perception phenomena such as the Helmholtz–Kohlrausch phenomenon [7, 65]. This corresponds to the fact that the quantum system of the rebit is closed, i.e., with no interactions with its environment. As opposite, open quantum systems may be interacting with other quantum systems as part of a larger system [66]. The resulting modification of the initial state space, i.e., of the space of density matrices, can be described by linear, trace-preserving, completely positive maps [67]. One may envisage explaining the phenomena mentioned above by such mechanisms.

An alternative approach to deal with context effects, based on the nonlocal theory of fiber bundles and connections, has been suggested by E. Provenzi in [68].

Finally, our work can be recasted in a much broader emerging field of research whose goal is to model general perceptual and cognitive phenomena from quantum theory, see for instance [69] or [70].

## Footnotes

1 That is the mathematical structure of the space of perceived colors.

2 It is tempting to draw parallels between this result and the more general point of view of [71].

3 We adopt here the notations of [17], although the spectral opponencies correspond in practice to the oppositions pinkish-red/cyan and violet/greenish-yellow. One can recover the oppositions red/green and blue/yellow with the multi-stage color model of R.L. and K.K. de Valois [16].

4 The usual way to derive the relativistic Lorentz transformations is to compute the linear transformations that leave invariant Minkowski’s quadratic form.

5 The boundary of *Ω* is supposed to be sufficiently regular.
